# Sexual attractiveness of male chemicals and vocalizations in mice

**DOI:** 10.3389/fnins.2014.00231

**Published:** 2014-08-05

**Authors:** Akari Asaba, Tatsuya Hattori, Kazutaka Mogi, Takefumi Kikusui

**Affiliations:** Department of Animal Science and Biotechnology, Graduate School of Veterinary Medicine, Azabu UniversityKanagawa, Japan

**Keywords:** mouse, pheromones, ultrasonic vocalizations, neural circuit, reproduction, multisensory integration

## Abstract

Male-female interaction is important for finding a suitable mating partner and for ensuring reproductive success. Male sexual signals such as pheromones transmit information and social and sexual status to females, and exert powerful effects on the mate preference and reproductive biology of females. Likewise, male vocalizations are attractive to females and enhance reproductive function in many animals. Interestingly, females' preference for male pheromones and vocalizations is associated with their genetic background, to avoid inbreeding. Moreover, based on acoustic cues, olfactory signals have significant effects on mate choice in mice, suggesting mate choice involves multisensory integration. In this review, we synopsize the effects of both olfactory and auditory cues on female behavior and neuroendocrine functions. We also discuss how these male signals are integrated and processed in the brain to regulate behavior and reproductive function.

## Introduction

Animals have evolved specific communication strategies to find a suitable mating partner and ensure reproductive success. These adaptive strategies are widely observed in the animal kingdom in a species-specific manner. In each species, sex-specific behavioral and sexual signals, such as chemical and auditory cues, transmit information about social and sexual status to the sexual counterpart, and this information is thought to be involved in mating success. In the majority of sexual encounters, females select their partners, or male compete with one another to obtain a chance to reproduce. The former is referred to as the “female mate choice.” This is probably because females have a smaller copulation potential and fewer reproductive outcomes than males and these sex biases in the level of parental investment create a condition that favors mating choice (Trivers, 1972). Therefore, male signals play a critical role in the females' choice of the best partner and in reproductive physiology. To date, many theoretical and empirical research studies have addressed the “good genes” hypothesis of mate choice, where females choose a mate based on the quality of genes that their offspring would inherit from the sire (Kokko et al., 2003; Neff and Pitcher, 2005; Andersson, 2006; Andersson and Simmons, 2006). In other words, a female's preference for male traits can provide genetic benefits to the offspring that inherit favorable alleles from the father (Mead and Arnold, 2004).

According to this hypothesis, three female strategies have been identified for mate preference. First, females prefer masculine males with a higher social rank to males with a lower social rank. Usually male social rank is related to circulating testosterone levels, with higher-ranking males having a higher testosterone level than lower-ranking males. Therefore, females prefer males with stronger testosterone-controlled signals, such as male chemosignals (Xiao et al., 2004), hair color (West and Packer, 2002), body masculinity (Roney and Simmons, 2008), and vocalizations (Pasch et al., 2011), which are also related to social rank. Second, females prefer healthy male individuals. For example, female mice can discriminate parasitized from unparasitized males via their odor (Kavaliers and Colwell, 1993), and are more attracted to the odor of unparasitized males (Kavaliers and Colwell, 1995a,b). Third, females prefer males that exhibit genetic-related signals that help them avoid inbreeding and increase genetic diversity, which also increases disease resistance (Potts et al., 1991; Penn and Potts, 1998b). Therefore, females receive variable information about the social and health status, and genetic background of individual males by the quality and quantity of their signals.

Furthermore, many reports show that male signals enhance reproductive function in recipient females. Especially, male mouse odors contain chemical cues called pheromones that exert powerful effects on the preference and reproductive biology of female mice. For example, in biological function, male urine is chiefly responsible for estrus synchronization (the Whitten effect; Whitten, 1958), puberty acceleration in young females (the Vandenberg effect; Vandenbergh, 1969), and pregnancy block (the Bruce effect; Bruce, 1959). In addition, male courtship vocalizations enhance reproductive function in avian and mammal species (Shelton, 1980; McComb, 1987; Leboucher et al., 1998; Delgadillo et al., 2012). Thus, male signals are used both as social cues for mate preference and as a way to stimulate female reproduction. This notion is logical because when a female encounters a “better male (masculine/dominant male),” she has to approach the male (preference) and copulate with him (reproduction) to increase her chances of obtaining offspring with greater fitness. It has actually been suggested that dominant males tend to sire more litters than subordinate males (DeFries and McClearn, 1970; Oakeshott, 1974), probably by the effects of dominant male signals in the preference and reproduction.

Male mice emit song-like “ultrasonic vocalizations (USVs)” both in the presence of females and when stimulated by a female's urinary pheromones (Nyby et al., 1977; Holy and Guo, 2005). Recent studies have revealed that USVs are attractive to female mice and that females prefer the USVs of males that are different from their parents (Hammerschmidt et al., 2009; Musolf et al., 2010; Asaba et al., 2014), similar to the pheromonal effects. Therefore, in a natural context, female mice usually use chemical and auditory cues that carry male-specific information required for mating. This means that laboratory mice are useful model for understanding female strategies of mate choice using multiple sensory stimulations. Here, we review discoveries about the effect of male pheromones and vocalizations on mate choice and their role in reproductive and neuroendocrine functions in female mice. Subsequently, we shed light on how multisensory stimuli from males are integrated in the brain and how this information regulates female behaviors and reproductive functions.

## Attractiveness of male pheromones

Females attend to male odors that contain various types of information, such as a male's dominance status. Dominance-associated traits in males, such as aggressiveness and territoriality, have long been viewed as ecologically significant. Since social subordinations suppress gonadal function in mice, emission of male-specific pheromones (Bronson, 1979), which are synthesized under the control of testosterone, are likely diminished in subordinates compared to those of dominant males. Actually, male social status is assessed through changes in testosterone-dependent volatiles in urine that are attractive to females (Jemiolo et al., 1985, 1991; Novotny et al., 1990). Moreover, peripubertal period (Postnatal day 21–38) exposure to male odors also accelerates the expression of mate odor preference in female (Jouhanneau et al., 2014).

The control of pheromones secretion by testosterone in the urine is not only for attracting females, but also for increasing reproductive function in females. Urine from dominant males is far more effective at promoting puberty acceleration in young female mice compared to urine from subordinate males and castrated males (Lombardi and Vandenbergh, 1977). Treatment with testosterone restored the ability of castrated males to induce these effects (Dominic, 1965; Lombardi et al., 1976), demonstrating that testosterone-dependent pheromones are responsible for female puberty acceleration. In addition, male pheromones have pivotal roles in inducing estrus synchronization (Novotny et al., 1990; Dulac and Torello, 2003) and terminating pregnancy in females (Bruce, 1960; Spironello Vella and deCatanzaro, 2001), whereas the urine from castrated males cannot induce these effects.

**Figure 1:**
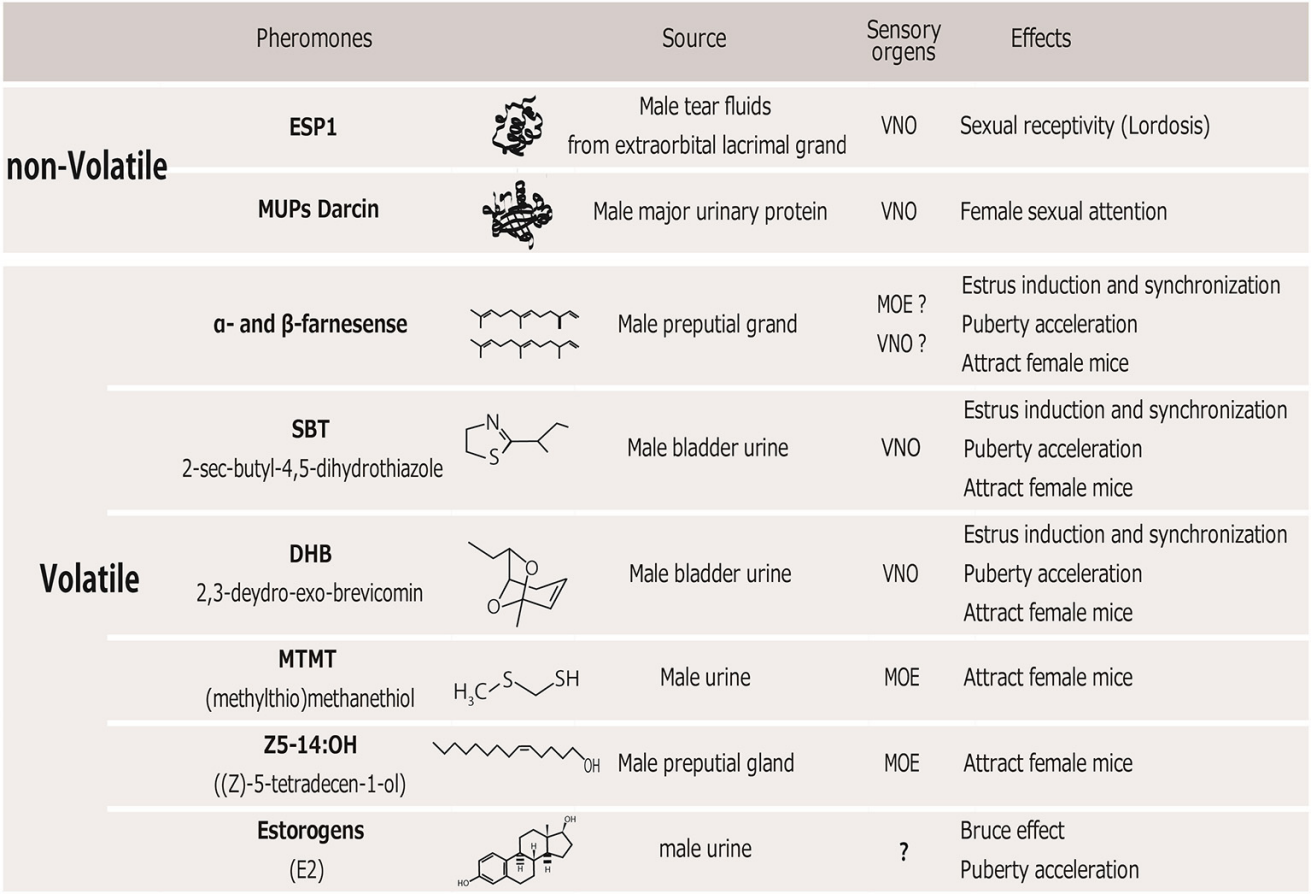
**Table of male pheromones that affect female mice**. VNO, vomeronasal organ; MOE, main olfactory epithelium.

Several instances have been identified in which a single pheromone compound can evoke certain effects on female sexual behaviors and reproduction as described above. These pheromones could be classically divided into “releaser pheromones,” which affect rapid mating behavior, and “primer pheromones,” which affect long-term physiological processes. Indeed, many identified pheromones have multiple behavioral and physiological functions (Novotny, 2003), suggesting that the distinction between releaser and primer pheromones is not meaningful at the stimulus level. Below, we review male pheromones that are reportedly attractive to females or that increase the reproductive function of female mice (Figure 1).

In rodents, pheromones are perceived by two separate olfactory systems, the main olfactory system (MOS) and the vomeronasal system (VNS), which convey chemical information to the central brain area. These two systems have separate sensory neural populations in the nasal cavity. It has traditionally been demonstrated that pheromonal molecules are mainly perceived through the vomeronasal organ (VNO) (Tirindelli et al., 2009). Non-volatile molecules contained in the urine or on the body's surface are transferred to the VNO when an animal's nares directly contact a stimulus on the opponent mouse. This behavior is commonly observed when mice show sexual or aggressive behavior. Therefore, the VNS is thought to mediate the detection of most species- and sex-specific cues involved in the control of mating and aggressive behavior (Stowers et al., 2002; Dulac and Torello, 2003). The MOS, on the other hand, mainly detects airborne scents, that is, volatile chemical components and small airborne peptides, via receptors in the main olfactory epithelium (MOE). Airborne volatiles are essential for detecting scents at a distance, which can alert animals to both the presence and location of a scent's source. In order to detect non- or low volatile components through the VNS, an animal must approach and make nasal contact with the source of the scent. In this review, we also describe the sensory organs responsible for detecting each of the identified compounds in the social odors (Figure 1) and their neural pathways below.

### Exocrine gland-secreting peptide1 (ESP1)

ESP1 is secreted into tear fluids from the extraorbital lacrimal glands (Kimoto et al., 2005) in adult male mice, and it promotes female sexual behaviors, such as lordosis (Haga et al., 2010). When female mice make close nasal contacts with either the facial area of an adult male or soiled bedding, non-volatile ESP1 binds to the specific vomeronasal receptor, V2Rp5, which is one of the V2R family receptor involved in female sexual behavior (Oboti et al., 2014). The ligand-receptor interaction results in sex-specific signal transmission through the accessory olfactory bulb (AOB) and medial amygdala (MeA), to the ventromedial hypothalamic nuclei (VMH), which serve as a center for female lordosis behavior (Haga et al., 2010).

### Darcin

The single atypical major urinary protein (MUP) named darcin is present in adult male urine (Roberts et al., 2010). Darcin can promote female attraction and induces the spatial learning of other chemical cues, allowing both females and competitor males to find sites of previous social interactions (Roberts et al., 2012). Because darcin is a non-volatile chemical like ESP1, female mice have to make direct contact with male urine to promote attraction to the chemicals and to enhance the conditioned place preference. Darcin may be perceived by the VNO, however, the receptors and neural pathways for darcin have not been identified.

### α- and β-farnesense

α- and β-farnesense induce territorial avoidance among male mice (Novotny et al., 1990; Jemiolo et al., 1992). The urine of dominant males has a higher concentration of these molecules compared to the urine of subordinate males. These molecules are conspicuously absent in bladder urine (Harvey et al., 1989), suggesting that α- and β-farnesense are synthesized in and secreted from the testes, or the preputial gland, probably by testosterone-dependent manner (Novotny et al., 1990). α- and β-farnesense also attract females (Jemiolo et al., 1991; Mucignat-Caretta et al., 1995), and can effectively induce estrus (Brennan and Peele, 2003; Dulac and Torello, 2003). These small ligands bind to MUPs in the urine, and this mixture shows puberty-accelerating pheromonal activity in recipient females (Novotny et al., 1999b). Thus, these molecules have complex functions in recipient females.

### 2-SEC-BUTYL-4,5-dihydrothiazole (SBT) and 3,4-deydro-exo-brevicomin (DHB)

SBT and DHB are the volatile urinary constituents in male mice; castration drastically reduces their concentration, while testosterone supplementation restores their concentrations to normal levels (Novotny et al., 1985). Although merely presenting a mixture of SBT and DHB has no primer pheromone effects on recipient females, the addition of these compounds to urine from castrated males can be attractive to females (Jemiolo et al., 1985) and can induce estrus synchronization (Jemiolo et al., 1986). It is possible that peptide molecules in male urine act as transporters that transmit SBT or DHB to the VNO, where these two compounds bind to receptors located in the vomeronasal epithelium. Actually, these compounds also bind to MUPs and increase puberty-accelerating pheromonal activity in recipient females (Novotny et al., 1999b).

### Other candidate male pheromones

(Methylthio)methanethiol (MTMT) is found in only male mouse urine and are attractive to females (Alema et al., 1988; Lin et al., 2005). The specific mitral cells in the main olfactory bulb (MOB) that respond to MTMT were not stimulated by any other urinary compound. Thus, MTMT is highly volatile, and it may advertise the presence of a male from a distance, perhaps as a signal to attract females. (Z)-5-tetradecen-1-ol (Z5-14:OH) was recently identified as a natural ligand for a mouse odorant receptor, and is excreted from the preputial gland into male mouse urine under the control of testosterone and enhances the attractiveness of urine to female mice (Yoshikawa et al., 2013). This compound could act as a natural ligand for the specific chemosensory receptor Olfr288 in the MOE.

### Estradiol (in male urine)

The occurrence of the Bruce effect and the Vandenberg effect in female mice is generally thought to depend on detection and processing of urinary odors from male through the VNS (Leinders-Zufall et al., 2004). Castration of male mice reduced these effects and treatment with either testosterone (Dominic, 1965; Lombardi et al., 1976) or estrogens (Thorpe and deCatanzaro, 2012) restores the ability of castrated males to induce these effects. Intact male mouse urine consistently contains substantial quantities of unconjugated estrogens, and the levels rise when males are in the presence of females (deCatanzaro et al., 2006). It was proposed that estradiol excreted in the male's urine may be ingested nasally by female (Guzzo et al., 2012; Baum and Bakker, 2013), and this circulating estradiol may directly influence blastocyst implantation (the Bruce effect) and maturation of the reproductive tract (the Vandenergh effect). Recently, it has been found that activation of VNO neurons was observed in response to sulfated steroids such as androgens, estrogens, and glucocorticoids (Nodari et al., 2008; Guzzo et al., 2010). Thus, estrogens in male urine can transmit information of the status of male mouse to females, acting either as a pheromone or as an endocrine stimulator. However, other female urine that also contains estradiol would not cause pregnancy block. Thus, some additional male pheromonal cues (such as MHC described below) plays a critical role in the Bruce effect.

## Pheromonal cues for avoiding inbreeding

Mice have a remarkable ability to detect individual genetic differences by their odors. In particular, mice can detect differences in urine odor type from mice with different major histocompatibility complexes (MHCs) under laboratory (Beauchamp et al., 1985; Penn and Potts, 1998b) and semi-natural conditions (Potts et al., 1991). The genes of the MHC are the most polymorphic coding loci known among vertebrates, and their products, MHC molecules, play a central role in immunological self/non-self recognition (Janeway et al., 2001). Interestingly, MHC genes also influence individual odor differences (Penn and Potts, 1998b). When given a choice via mating or two-choice assays, female mice prefer males with MHCs that are different from their own (Yamazaki et al., 1976). It has been suggested that the peptide ligand of MHC class I molecules are release into urine and can elicit an MHC-haplotype-specific behavioral response after uptake into the nose by sniffing (Overath et al., 2014). As a result, mice can increase the MHC-heterozygosity of their progeny. MHC-dependent mating preferences may function to increase disease resistance, since MHC-heterozygous offspring have less inbreeding and fewer hereditary diseases (Potts and Wakeland, 1993; Brown and Eklund, 1994; Apanius et al., 1997). Interestingly, MHC-related chemicals may be responsible for the Bruce effect (Leinders-Zufall et al., 2004). MHC-related chemicals are important for forming olfactory memories in the context of pregnancy block.

Recent studies revealed that females also use variant MUP profiles to recognize the urine scent marks of individual males (Hurst et al., 2005; Cheetham et al., 2007). These MUP patterns in the urine act like an individual “bar code” that signals the identity of the scent marks' owner (Beynon and Hurst, 2003). In wild house mice, females use self-referent matching of MUP patterns to avoid inbreeding, but there is little evidence that MHC sharing influences mate choice (Sherborne et al., 2007). Indeed, MUPs have been implicated as chemical signals that allow the recognition of genetic heterozygosity (a sign of phenotypic vigor) and enable the avoidance of inbreeding. Furthermore, MUPs help distinguish individuals of the same or different species.

Collectively, it appears that MHC-related chemicals and MUPs contribute non-volatile proteins or peptides of mouse scent signals, permitting individual recognition, for which mice have specific receptors in the VNO (Leinders-Zufall et al., 2004). MUPs show individual expression consistent with a role in sexual signaling, and these MHC and MUP signals may provide information regarding an individual's genetic identity that could be used in mate selection.

## Pheromonal effects on female neuroendocrine function

As mentioned above, male pheromones can modulate female behaviors, but they also stimulate female reproductive abilities. Regulating these reproductive effects involves male pheromone signatures, which are formed in part by neural circuitry within the olfactory system and hypothalamic-pituitary-gonadal (HPG) axis. Modification of the HPG axis, which is regulated by male pheromones, stimulates the release of luteinizing hormone (LH) and prolactin (PRL), two hormones that are important for female reproductive function (Halpern, 1987; Mak et al., 2007). These pituitary hormones subsequently act on the gonads. Actually, gonadotropin-releasing hormone (GnRH) neurons, which are the key hormone regulators of LH secretion, originate in the olfactory system in the early neonatal period, and migrate into the hypothalamus, suggesting a tight connection between olfaction and reproduction (Wray, 2010). In fact, pheromones from opposite sex conspecifics induce sexually dimorphic responses in GnRH neurons in mice (Yoon et al., 2005), and, neuropeptide kisspeptin which play an important role in modulating GnRH neurons, are activated by male odor in female mice (Bakker et al., 2010). Compared to the MOS, the VNS provides a neural pathway that directly links olfactory sensations to the hypothalamus, and the modulation of LH and PRL release through this pathway seems to provide the endocrine basis for the “primer” effect of pheromones.

These hypothalamic areas are involved in reproductive endocrinology and sexual behavior in females. In particular, the GnRH-LH axis has a key role in the “releaser” effect, in that it regulates pheromone-mediated sexual behavior. GnRH itself can reverse the effects induced by surgical removal of the VNO, which diminishes sexual behavior, including mount attempts in males (Fernandez-Fewell and Meredith, 1995) and lordosis responses in females, highlighting the crucial role of this hormone in the regulation of female sexual behavior (Mackay-Sim and Rose, 1986). Therefore, pheromones can enhance sexual behavior via stimulating GnRH release. Another pathway for modulating sexual behavior is through the activation of the HPG axis. Explicitly, primer pheromones increase activity of the GnRH-LH axis, which stimulates estrogen release from the ovaries (Kerbeshian et al., 1994). Circulating gonadal hormones, especially estradiol and progesterone, enhance female sexual behavior (Lydon et al., 1995), and probably exert rapid non-genomic effects. Specifically, estrogen can rapidly stimulate GnRH neurons in the preoptic area (POA) via estrogen receptor β (ERβ), implying that either circulating estrogen or *de novo* synthesized estrogen in the brain can rapidly stimulate female sexual behavior through GnRH neurons. There is no clear evidence showing that pheromones induce *de novo* estrogen synthesis in the hypothalamus, thus future research should establish whether male pheromones are able to enhance *de novo* synthesis of estrogen in the POA via stimulating aromatase in the neurons.

## Attractiveness of male vocalizations

Females might use vocalization to assist in mate selection (McComb, 1991; Rehsteiner et al., 1998). In many species, vocal displays associated with sexual encounters are often mediated by testosterone released from the testes (Floody, 1981; Moore et al., 2005; Bass and Remage-Healey, 2008). Thus, testosterone levels strongly influence vocal performance in some species because vocalizations accurately reflect a male's social status and resource holding potential (Parker, 1974; Galeotti et al., 1997). For example, in birds, males that had calls with high vocal performance scores were considered more successful breeders than males with low performance scores (Ballentine et al., 2004; Illes et al., 2006; Janicke et al., 2008). Similarly, in deer, there is strong relationship between the time invested in vocal display and their mating success (McElligott et al., 1999).

Male mouse USVs are also under androgenic control (Dizinno and Whitney, 1977; Nunez et al., 1978; James et al., 2006). Female mice move toward male USVs, suggesting that the USVs of male mice are attractive to female mice (Hammerschmidt et al., 2009). Likewise, in neotropical singing mice, testosterone plays a strong role in modulating vocal performance in males, increasing the duration and complexity of trills (Pasch et al., 2011). Females in neotropical singing mice spend more time near speakers that generate high-performance trills resembling the vocalizations of testosterone-treated males (Pasch et al., 2011). Moreover, dominant males emit more USVs toward a female than male of lower rank in C57/BL6 mice (Wang et al., 2011). Thus, testosterone levels strongly influence vocal performance because vocalizations reflect a male's social status in mice. These findings indicate that the vocalization of male mice function to show females their masculine phenotype, which is under the control of testosterone, similar to the vocalization of other species.

## Male vocalizations for avoiding inbreeding

The characteristics of male mouse USVs differ across inbred strains (Panksepp et al., 2007; Kikusui et al., 2011; Sugimoto et al., 2011). A cross-fostering study revealed that the sequences of male USVs are under strong genetic control (Kikusui et al., 2011). Research using transgenic mouse models suggests that USV profiles are genetically regulated in males (Wang et al., 2008; Ey et al., 2012; Hammerschmidt et al., 2012; Roy et al., 2012; Mahrt et al., 2013). Although the song characteristics of male USVs are highly variable, the biological significance of these repertoires has not been identified. To demonstrate that female mice can discriminate different male USVs, we recently conducted playback experiments to assess the responses of female mice to USVs of male mice from different strains. We found that inbred female mice could discriminate the different male USV characteristics, and that they preferred the USVs of mice that were from different strains than their parents (Asaba et al., 2014). Similarly, wild-derived female mice showed a greater preference for USVs produced by unfamiliar males compared to the USVs produced by familiar males (Musolf et al., 2010; Hoffmann et al., 2012), implying that female mice can use male USVs to select a mating partner.

As mentioned above, a cross-fostering study in male mice revealed that USV sequences are under strong genetic control (Kikusui et al., 2011). It is of interest whether the preference for male USVs in females is genetically controlled or experience-dependent. We conducted the same cross fostering experiment in female mice and found that these preferences are based on early social experiences, unlike the male USV characteristics, which are genetically controlled (Asaba et al., 2014).

These findings suggest that the preference for USVs of males from a different strain contributes to disassortative mating, which is an important mate choice strategy for avoiding inbreeding and facilitating the heterozygosity of offspring. As described above, MHCs and MUPs have a pivotal role in disassortative mating, in that mice avoid individuals with similar MHCs and MUPs using chemical cues. Our recent findings suggest that another social signal, male mouse songs, have an important role in courtship by potentially facilitating mate attraction or mate choice.

## Male vocalizations enhance female estrogens and mating behavior

Although it is unclear whether the neural circuits for ultrasonic songs are responsible for reproductive outcomes in mice, other reports show that male courtship vocalizations trigger female sexual and endocrine responses in avian and mammal species. The effects of male vocalizations on female reproduction are well documented in songbirds. Songs presented to females via audio playback are sufficient to enhance female reproduction, such as LH secretion, follicle growth, egg laying, and nest-building behavior (Brockway, 1965; Kroodsma, 1976; Hinde and Steele, 1978; Morton et al., 1985; Leboucher et al., 1998; Bentley et al., 2000). Additionally, in mammals, playback vocalizations of males advance the onset of seasonal ovulatory activity in red deer (McComb, 1987). Similarly, the auditory signals emitted by bucks are strong enough to stimulate the secretion of LH from the pituitary, sexual behavior, and ovulation in female goats (Shelton, 1980; Delgadillo et al., 2012). These reproductive effects induced by male vocalizations are mediated by the development of ovarian follicles and the consequent production of estradiol, which is a key hormone for displaying sexual behavior.

## Multisensory integration for female behavior

The social environments of animals are very complex, and correspondingly place great information processing demands on the brain. Individuals are faced with complex and multisensory signals from which they have to extract functionally relevant cues. As mentioned above, there is behavioral evidence that supports the value of each sensory signal separately, but how these sensory signals are integrated in a natural context has not been elucidated. In male-female communication, it is normal for females to be simultaneously exposed to olfactory and acoustic cues emitted by males. One possible mechanism for sensory integration is that the exposure of female mice to chemical and acoustic cues from males activates a common neural circuit involved in reproductive function and behavior.

Earlier work has shown that adult females have an innate interest in male USVs (Pomerantz et al., 1983), but that females habituate rapidly to the pure playback of male USVs in the absence of real males (Hammerschmidt et al., 2009; Shepard and Liu, 2011). When females were exposed to living males, they showed a reinstated preference for these vocalizations upon retest, suggesting that chemical or physical contact with males is important for maintaining the response to vocal cues (Shepard and Liu, 2011). Behaviorally, female mice discriminate among males not only using olfactory cues, but also using USVs, and it has been shown that females prefer unfamiliar individuals to familiar kin (Bowers and Alexander, 1967; Hammerschmidt et al., 2009; Musolf et al., 2010; Shepard and Liu, 2011). We demonstrated that olfactory signals have significant effects on mate choice when paired with acoustic cues in mice. The preference for USVs appeared when females were exposed to male-soiled bedding or to ESP1 before USV preference tests (Asaba et al., 2014). Interestingly, both auditory (USV) and odorant (MHC) preferences for mate choice were imprinted during the developmental period of female mice (Penn and Potts, 1998a). This implies that the integrated sensory system for mate choice has plastic qualities that are dependent upon social experiences during the pre-weaning period.

Multisensory information is also important for inducing maternal behavior in mother-infant communication (Levy and Keller, 2009). Maternal retrieving behavior is regulated by multisensory information from pup olfactory and auditory cues (Okabe et al., 2013). In electrophysiological studies, exposure to pup odors enhances the neuronal responses to pup USVs in the auditory cortex in the mother, indicating that sensory information from the pup, including both acoustic and chemical signals, affects the function of the auditory cortex (AuC) (Cohen et al., 2011). It is hypothesized that male odors enhance the processing circuits for acoustic information, or vice versa, which may be the neural mechanisms underlying female mate choice. Multimodal sensory systems for mate choice may explain the cross-modal information processing involved in this behavior. Below, we provide a detailed examination of the possible neural circuits involved in the sensory integration of chemical and audible cues.

## Olfactory pathway

**Figure 2:**
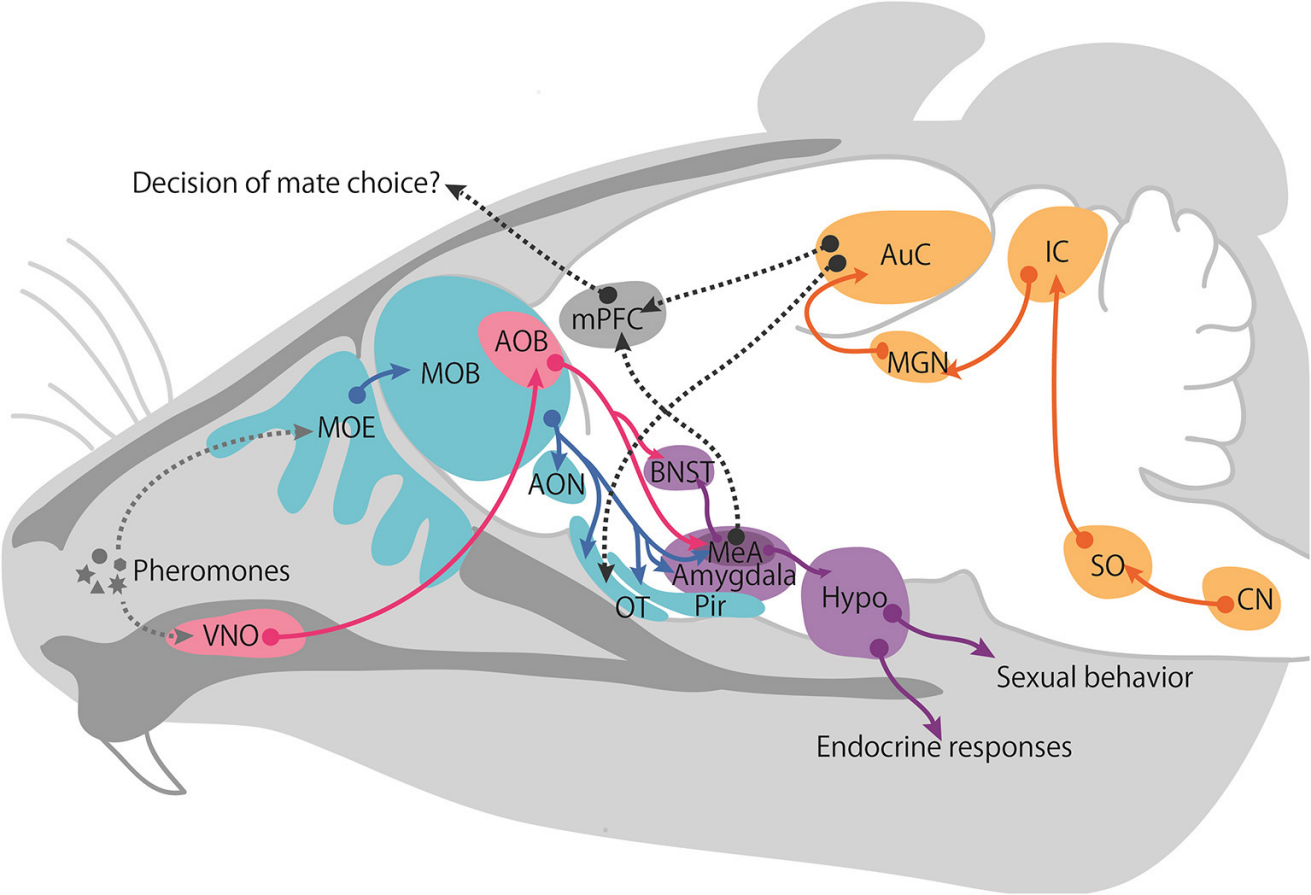
**Proposed schematic of female neural pathways for male chemical and vocalization signals**. The vomeronasal pathway is shown in pink, main olfactory pathway in blue, intracortical projection in purple, auditory pathway in yellow, and the hypothetical integrate pathway is represented with a gray dotted line. Abbreviations: accessory olfactory bulb (AOB), anterior olfactory nucleus (AON), auditory cortex (AuC), bed nucleus of the stria terminalis (BNST), cochlear nucleus (CN), Hypothalamus (Hypo), inferior colliculus (IC), medial amygdala (MeA), medial geniculate nucleus (MGN), main olfactory bulb (MOB), main olfactory epithelium (MOE), medial prefrontal cortex (mPFC), olfactory tubercle (OT), piriform cortex (Pir), superior olivary nucleus (SO), vomeronasal organ (VNO).

While no behavioral or neural evidence supports the existence of multimodal sensory processing in mice, the basic anatomy and physiology of the pheromone-processing circuits are well defined and available. The two major olfactory systems, the MOS and VNS, convey chemical information to the central nervous system (Figure 2). VNO receptors extend axons to glomeruli located in the AOB. AOB mitral cells, which are second-order neurons, extend axons to the MeA (Kevetter and Winans, 1981). In turn, neurons in the MeA project to hypothalamic targets that control female proceptive (approach) and receptive (lordosis) behaviors (Brennan and Zufall, 2006). Therefore, one characteristic of the VNS is that it sends information directly to the behavioral and endocrine center of the hypothalamus, which is not involved in the cortex-thalamic sensory processing pathways.

In contrast, the MOS has traditionally been recognized as the detection system for non-pheromonal odorants present in the environment. As mentioned earlier, the MOS detects airborne scents, that is, volatile chemical components and small airborne peptides, via receptors in the MOE, and these scents can be detected at some distance from their source. Receptor neurons in the MOE extend axons to mitral cells in the MOB. The mitral cells of the MOB project to several higher centers including the anterior olfactory nucleus (AON), the olfactory tubercle (OT), the piriform cortex (Pir), and the cortical amygdala (Kevetter and Winans, 1981). Recently, studies have shown that a subpopulation of MOB mitral cells projects directly to the MeA (Pro-Sistiaga et al., 2007; Kang et al., 2009; Thompson et al., 2012). The AOB and MOB pathways converge in several cortical and sub-cortical amygdaloid targets, suggesting that these locations are where chemosignals involved in social communication are integrated.

Although the MOS is thought to detect volatile odorants and the VNS is thought to be specialized for the detection of non-volatile pheromones, recent findings suggest that the MOS is also involved in pheromone detection (Alema et al., 1988; Boehm et al., 2005; Lin et al., 2005; Yoon et al., 2005; Spehr et al., 2006). As mentioned above, adult male mice produce several male-specific volatiles in their urine under the control of testosterone (Schwende et al., 1986; Alema et al., 1988; Novotny et al., 1999a; Lin et al., 2005), and these chemicals can be detected through the MOE and VNO. Therefore, the recognition and assessment of conspecifics though scents involves an important interaction between the MOS and VNS that controls sexual attraction. A functional magnetic resonance imaging (fMRI) study revealed that MOB activation in female mice occurred slightly earlier than activation in the AOB in response to male urinary volatiles (Xu et al., 2005). These results suggest that the VNO responses to volatile pheromones are typically preceded by MOE detection, which then leads the animal to make nasal contact with a non-volatile pheromone, thus activating the VNO. Once animals are in close nasal contact with the scent source, the VNO pump is activated to gain additional information through the VNS. Therefore, detecting airborne scents through the MOS may be necessary to activate scent delivery to the VNO (Hurst and Beynon, 2004; Keller et al., 2006).

Once the sex information of an individual is encoded, it is transmitted to the limbic system. The MeA in the limbic system is one of the most important regions for integrating this information since it receives axons from both the MOS and VNS (Pro-Sistiaga et al., 2007; Kang et al., 2009; Thompson et al., 2012). The MeA sends the information to the hypothalamic nuclei, including the VMH and bed nucleus of the stria terminalis (BNST), both of which are involved in sexual or aggressive behavior (Brennan and Zufall, 2006). Therefore, the chemical information within social stimuli perceived by the MOS and VNS acts in simple and stereotyped neural circuits that lead to behavioral and neuroendocrinological outcomes.

## Estrogen modulates chemical neural pathways

Olfactory sensitivity and preferences are modulated by sex hormones. The neural mechanisms underlying the action of estrogens on the olfactory system are intricately organized. Studies in humans suggest that estrogens enhance olfactory sensitivity by acting on the reception and perception of chemicals. For example, the composition of mucus in the olfactory epithelium is altered by the menstrual cycle (Mair et al., 1978). Similarly, estrogens enhance olfactory sensitivity and olfactory preferences through peripheral and central mechanisms in rodents (Pietras and Moulton, 1974; Moffatt, 2003). In fact, the olfactory sensitivity of female mice changes throughout the estrous cycle, and ovariectomized animals are sensitive to treatment with exogenous steroids (Caroom and Bronson, 1971). Indeed, after exposing estradiol-treated females to soiled male bedding, more VNO neurons were activated in estradiol-treated females compared to in oil-treated females (Halem et al., 1999). However, male urine activated mitral and granule cells in the AOB of female mice independently of estrogens. In the central portion of the VNS, namely the BNST and medial POA, neuronal cFos responses to male pheromones, an indicator of neural activation, were observed equally in females regardless of estrogen treatment, implying that estrogen effects mainly occur in VNO sensory neurons. Therefore, the effects of estrogens on the sensitivity to male pheromones and on neuronal responses to male pheromones probably work in concert to maximize the likelihood of mating and reproduction in reproductively permissive environments (Moffatt, 2003).

Steroid hormone receptors, especially estrogen receptors, are widely distributed in the MOS and VNS. Estrogen receptor α (ERα) is located in the MOB and AOB (Merchenthaler et al., 2004). In the subsequent region that receive axons from the MOB and AOB, both ERα and ERβ are expressed in the cortical and medial divisions of the amygdala, which is the key brain region involved in the integration of chemical information from the MOB and AOB (Mitra et al., 2003). ERα and ERβ are also found in the POA and BNST, suggesting that estrogens can act on the central neural circuits of olfaction. Interestingly, the distribution of estradiol-induced progestin receptors is similar to ERs, and estradiol-induced progestin receptors colocalize with ERs in the amygdala, hypothalamus, and POA (Moffatt et al., 1998). Regarding female sexual behavior, combined treatment with estrogen and progesterone enhances female lordosis behavior (Pfaff, 1994); therefore, neurons in the olfactory pathway that express ERs and progesterone receptors can be involved in female sexual behavior induced by chemical stimulation.

## Auditory pathway

Regarding ultrasonic neural circuits in mice, it is not clear which neural circuits are responsible for the behavioral and reproductive outcomes. However, neural circuits for audible sounds have been identified. Encoded sound information is transmitted from the cochlea to the central nervous system (Figure 2). The auditory nerve enters the brainstem at the pons-medulla junction and sends its axons to the cochlear nucleus (CN). The secondary auditory neurons in the CN send their axons decussately in the ventral pons and ascend to the superior olivary complex (SO). The SO is important for detecting the interaural level and time differences necessary for sound localization. Third-order neurons of the SO send axons via the lateral lemniscus to the inferior colliculus (IC) of the midbrain, which is important for binaural information processing and is a major site of auditory information integration. Fourth-order neurons continue to the medial geniculate body (MGN), which is the thalamic relay where sensory information is filtered before it is transmitted to the cortex. The axons of fifth-order MGN neurons synapse with neurons of the AuC on the superior temporal gyrus (Charitidi, 2011). Given that the primary AuC is involved in auditory object recognition and is a known site of neuronal plasticity (Nelken, 2004; Weinberger, 2004; Nelken and Bar-Yosef, 2008; Miranda and Liu, 2009; Romanski and Averbeck, 2009), the learning and memory of vocalizations likely occur in this region.

Although the evolution of male courtship signals and the corresponding sensitivity of auditory responses in females are found in a variety of species (White et al., 1992; Vyas et al., 2009), there is no clear evidence pinpointing the neural circuits responsible for the female preference for male ultrasonic songs in mice. Furthermore, less is known about how neurons respond to multisensory stimuli during the imprinting of female preference for mate choice. A recent study showed that neural activity in the mouse OT regarding MOS is also responsive to auditory input, that is, a subpopulation of neurons in the OT displayed responses to a 2.8-kHz pure tone (Wesson and Wilson, 2010). Anatomically, neurons in the primary AuC send fibers to the olfactory Pir, indicating that auditory sensory information is transmitted to the olfactory cortex (Budinger et al., 2006; Budinger and Scheich, 2009). However, these studies used artificial pure tones, thus how natural male signals merge remains unclear.

## Estrogen modulates auditory neural pathways

Estrogens also modulate the physiological and reproductive behavioral responses to sensory signals, including auditory stimuli, in many species (Maney and Pinaud, 2011). For example, in humans, fluctuations in auditory perception (Haggard and Gaston, 1978; Cowell et al., 2011) and in electrophysiological measures of auditory function (Walpurger et al., 2004; Al-Mana et al., 2010) during the menstrual cycle highlight the effects of estrogen on auditory processing. When estradiol levels are exogenously modified, alterations in auditory perception, auditory brainstem latencies, auditory thresholds, and sound localization are observed (Haggard and Gaston, 1978; Jerger and Johnson, 1988; Wharton and Church, 1990; Coleman et al., 1994; Caruso et al., 2003). Direct evidence from animal electrophysiological and behavioral studies shows that hearing plasticity and communication skills are enhanced by estradiol (Sisneros et al., 2004; Remage-Healey et al., 2008, 2012; Tremere et al., 2009). Estradiol can rapidly modulate neural responses in the auditory association cortex of the zebra finch by suppressing inhibitory transmission (Tremere et al., 2009). Additionally, in mice, estrogen antagonists alter the auditory feedback mechanisms in mice (Thompson et al., 2006).

These estrogen effects are mediated by both ERα and ERβ, which belong to the nuclear receptor superfamily and act as transcription factors. Regarding auditory processing circuits, both ERα and ERβ are expressed in the peripheral organs and central auditory systems in mice (Stenberg et al., 1999; Charitidi and Canlon, 2010; Charitidi et al., 2010). Specifically, ERα and ERβ were found predominantly in the CN, the nucleus of the trapezoid body, the lateral- and medio-ventral periolivary nuclei, the dorsal lateral lemniscus, and the IC.

However, it is not evident whether male mice USVs play a role in female reproductive function or not; several studies revealed that estradiol is necessary for reacting to vocalizations. In mice, female preference for the vocalizing male was absent after ovariectomy, and was recovered via treatment with ovarian hormones (Pomerantz et al., 1983), indicating that female USV preference depends on the gonadal estrogens. Previous studies using a choice test showed that female USV preference was not altered by the estrus state (Hammerschmidt et al., 2009; Shepard and Liu, 2011); however, our recent study demonstrated that the preference for male USVs from a different mouse strain depended on the phase of the estrus cycle (Asaba et al., 2014). These results indicate that a certain level of estrogen is necessary for eliciting female preference for male vocalizations. The mechanisms and neural circuits underlying the role of estrogen in song preference, including where and how it acts on neurons, is the target of future research.

## Neural mechanisms for mate choice

In humans and non-human primates, the anterior medial prefrontal cortex (mPFC) and rostral anterior cingulate cortex (ACC) are related to certain components of social interpretation and behavioral interaction (Damasio, 1996; Stuss et al., 2002; Adolphs, 2009). These brain regions are active during judgments of kinship and close others later in life (Krienen et al., 2010). Regarding auditory preference, the choice of listening to one type of music over another likely reflects limbic, decision-making brain regions, such as the prefrontal cortex in humans (Levitin and Tirovolas, 2009). In rodent models, the lesion studies suggest that regions along the frontal midline including the mPFC and ACC have a critical role in behavioral decision-making (Kvitsiani et al., 2013). Interestingly, Jouhaneau and Bagady (1984) demonstrated that Swiss albino mice exposed to a certain type of music during postnatal days 10–20 demonstrated a preference for that kind of music when given a choice during adulthood, indicating that there is a sensitive period for forming auditory preferences even in mice. Moreover, the acoustic environment during the critical period for auditory preference shapes the mPFC response in C57BL/6 mice. Furthermore, this preference can be altered by epigenetic modification to the mPFC (Yang et al., 2012), suggesting that the mPFC has a pivotal role in imprinting auditory preferences in mice. We recently found that the female preference for male USVs is formed during the juvenile period while in the presence of the father mouse (Asaba et al., 2014). Therefore, the mPFC may be responsible for the plasticity of the vocalization preference in female mice.

In contrast, there is little evidence showing mPFC involvement in pheromone-induced mate preference. As mentioned above, chemical information perceived in the VNO is transmitted to the MeA and ends in the hypothalamus. Therefore, there is no direct connection between the VNS and mPFC. However, even in the pheromonal circuits underlying the olfactory-limbic-hypothalamus pathway, there is anatomical evidence of a connection between the amygdala and prefrontal cortex (McDonald et al., 1996; Marek et al., 2013), especially for emotional behavior.

There is one example of prefrontal cortex involvement in odorant preference. The male-derived volatiles become attractive if they are associated with non-volatile attractive pheromones, suggesting that non-volatile pheromones can stimulate the reward circuit via the VNS and act as an unconditioned stimulus. When female mice were exposed to the non-volatile pheromones, the basolateral amygdala and the shell of the nucleus accumbens were activated. In contrast, exploring the volatile pheromones conditioned with non-volatile pheromones activated the basolateral amygdala, prefrontal cortex, and ventral tegmental area (Moncho-Bogani et al., 2005). Therefore, the prefrontal cortex can be involved in the reward circuit via stimulation by male pheromones in females, resulting in female mate-preference behavior (McDonald et al., 1996; Moncho-Bogani et al., 2005). Collectively, one feasible neural mechanism for the sensory integration of chemical and auditory cues involved in female mate preference is the mPFC, which is activated by both pheromones and auditory cues. Future studies are needed to clarify this hypothesis.

## Summary

**Figure 3:**
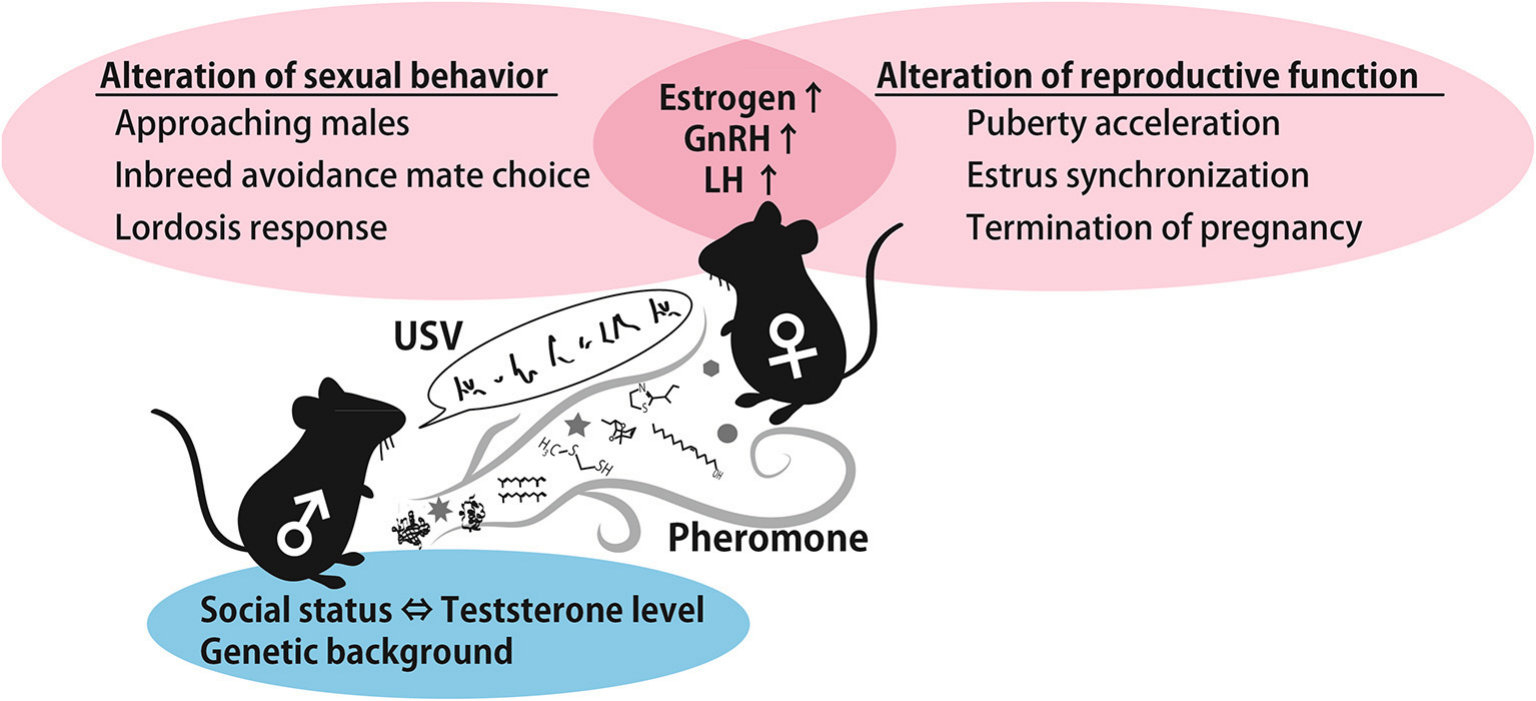
**A schematic illustration of the effect of male cues**. Testosterone-controlled male pheromones alter female sexual behavior and reproductive function by increasing estrogen and GnRH. In addition, genetic background-dependent male USVs and pheromones contribute to inbreeding avoidance. Abbreviations: gonadotropin-releasing hormone (GnRH), luteinizing hormone (LH), ultrasonic vocalization (USV).

As described above, female preference for male traits can provide genetic benefits to offspring that inherit favorable alleles from their father. Therefore, female reproductive functions can be activated if the female encounters a masculine male in order to obtain offspring with greater fitness. It is generally agreed that dominant males tend to sire more litters than subordinate males (DeFries and McClearn, 1970; Oakeshott, 1974). Female usually use pheromones and ultrasonic vocalizations, and these signals carry the male-specific information required to support these phenomena. The summarized roles of pheromones and USVs in males to female cues are shown in Figure 3. Although it is unclear which neural circuits are involved in USVs preference and the stimulation of reproductive outcomes in females, the behavioral and reproductive effect of pheromones and vocalizations are similar, suggesting that a common neural pathway integrates both types of information. Moreover, both signals characteristically contribute to inbreeding avoidance by imprinting during the developmental period. Imprinted sound preference is associated with mPFC activation in mice, as is a conditioned preference to pheromones. These results imply that male signals are integrated and processed in the mPFC to regulate behavioral decision-making processes. This idea is schematically represented in Figure 2. Understanding how females integrate sensory information from males, that is, pheromonal and auditory cues, in natural settings from a neuroscience and adaptive behavior perspective is of great interest.

### Conflict of interest statement

The authors declare that the research was conducted in the absence of any commercial or financial relationships that could be construed as a potential conflict of interest.
